# Metagenomic Next-Generation Sequencing to Investigate Infectious Endophthalmitis of Brucella: A Case Report

**DOI:** 10.3389/fmed.2022.847143

**Published:** 2022-03-29

**Authors:** Huiyu Xi, Lishuai Zhang, Bo Xu, Haiyang Liu, Suyan Li

**Affiliations:** Department of Ophthalmology, The Affiliated Xuzhou Municipal Hospital of Xuzhou Medical University, Xuzhou First People’s Hospital, Xuzhou Eye Disease Prevention and Treatment Institute, Xuzhou, China

**Keywords:** infectious endophthalmitis, high-throughput sequencing, brucella, ocular, metagenomic next-generation sequencing

## Abstract

**Introduction:**

Brucellosis is a systemic disease that exists prevalently in clinical manifestations. The symptoms present in organs such as the eyes (in ocular brucellosis) can lead to misdiagnosis or even failure to diagnose. Metagenomic Next-Generation Sequencing (mNGS), a high-throughput sequencing approach, could be applied for the detection of microorganisms.

**Case Presentation:**

A 57-year-old female with acute right-eye vision loss, treated with clindamycin and dexamethasone sodium phosphate for 1.5 months, was difficult to diagnose using regular methods. mNGS was utilized for the aqueous fluid from the patient, and Brucella melitensis was identified. The inflammation was treated with 3 months of antibiotherapy. However, even with specific medicine and surgery, the vision remained poor because severe ocular conditions last for a long time.

**Conclusion:**

It suggests that brucella should still be a probable pathogen in endophthalmitis despite its low incidence in non-epidemic areas. Moreover, mNGS can achieve early diagnosis and timely treatment for difficult-to-diagnose ocular infections.

## Introduction

Brucellosis, a zoonotic disease that is caused by brucella, exists around the world (1). Although the cases of Brucellosis have been greatly reduced in recent decades, it is notably present in many developing countries due to a series of recognized complications (2–4). Patients get infected by direct or indirect contact with brucella, such as *via* infected animals or ingestion of uncooked meat. Brucella spreads over damaged skin and mucous membranes through the digestive or respiratory tract. Brucellosis is thus a systemic disease that involves organs or systems of the body. Chronic brucella can be avoided through timely diagnosis and treatment; otherwise, it can lead to conditions that are severely debilitating and disabling. Acute phase cases mainly present with fever, fatigue, hyperhidrosis, muscle and joint pain, and swelling of the liver, spleen, and lymph nodes. Chronic phase cases often present with joint damage (5–7).

Herein, we report a case of infectious endophthalmitis caused by *Brucella melitensis*. The diagnosis is attributed to Metagenomic Next-Generation Sequencing (mNGS) technology, which provides information on the genomic sequences of microorganisms (8, 9). This unbiased high-throughput sequencing approach could identify the total DNA or RNA content of all currently known pathogenic microorganisms by performing simultaneous and independent sequencing of thousands to billions of DNA fragments (10).

## Case Description

**FIGURE 1:**
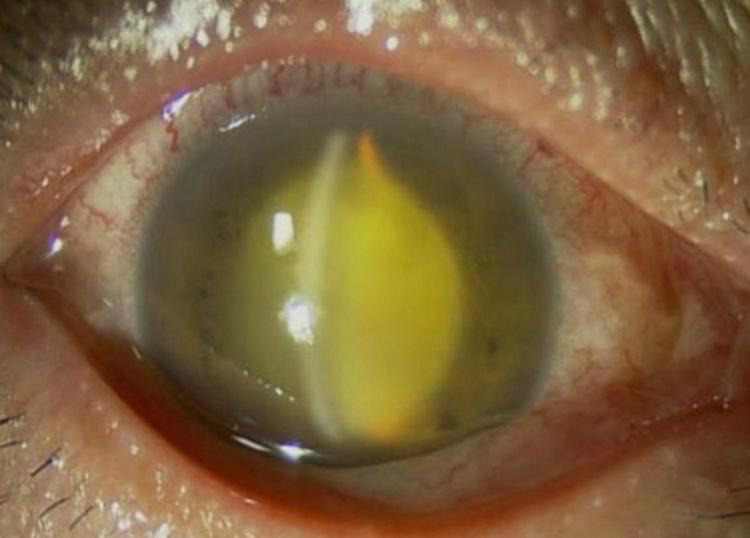
Anterior segment photograph of the right eye shows slight hyperemia of the conjunctiva, transparent cornea, deep anterior chamber, turbid aqueous humor, less round pupil, posterior iris adhesion, opaque lens, obvious vitreous opacity, with fundus invisible.

**FIGURE 2:**
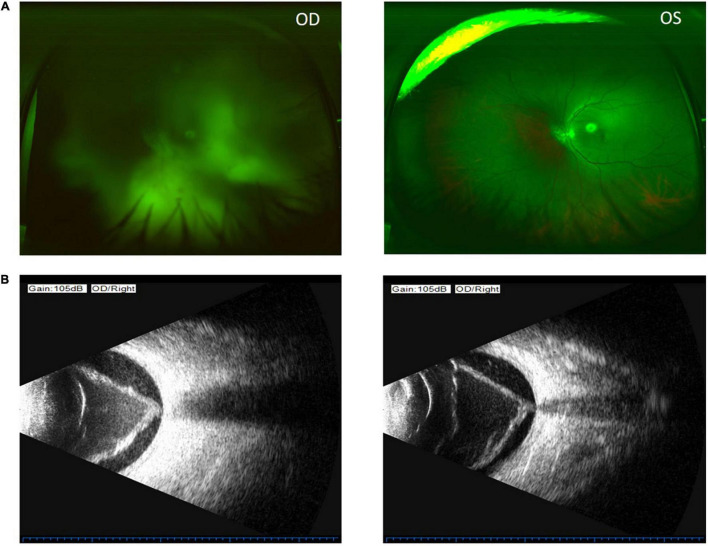
Color funds photograph shows invisible fundus of the right eye (upper left) and no fundus pathology of the left eye (upper right) **(A)**. Ocular B-ultrasound of the right eye shows vitreous opacity, vitreous membranes, and retinal detachment in the right eye (lower left and lower right) **(B)**.

On 13 August 2020, a 57-year-old female was admitted to the hospital due to right-eye vision loss for 1.5 months. Previously, the patient had received treatment in a regional hospital on 21 June. She was diagnosed with a vitreous hemorrhage and was treated with oral medications. The patient developed a fever with red eyes and eyelid edema in the right eye 2 days later. Positive symptoms of the right eye including mixed conjunctival hyperemia, retrocorneal pigment KP, aqueous turbidity, and exudation in the pupil area, and these were recorded by the regional hospital. The structure of the posterior segment of the eye is unclear. The local ophthalmologist diagnosed panuveitis of the right eye and applied mydriatic drugs, anti-inflammatory dexamethasone eye drops, and intravenous infusion of clindamycin and dexamethasone sodium phosphate 10 mg. After these treatments, the patient felt the symptoms lessened, and the systemic dexamethasone sodium phosphate was thus gradually reduced to 5 mg. However, the eye symptoms were then aggravated. The patient was transferred to a tertiary hospital afterward. Her initial visual acuity was Hand Motion OD and 20/30 OS. Slit-lamp examination revealed slight hyperemia of the conjunctiva, transparent cornea, deep anterior chamber, turbid aqueous humor, less round pupil, posterior iris adhesion, opaque lens, and obvious vitreous opacity with invisible fundus of the right eye (Figure 1). There was no ocular pathology of the left eye. The patient was diagnosed with infectious endophthalmitis and admitted to the current hospital (Figure 2).

**FIGURE 3:**
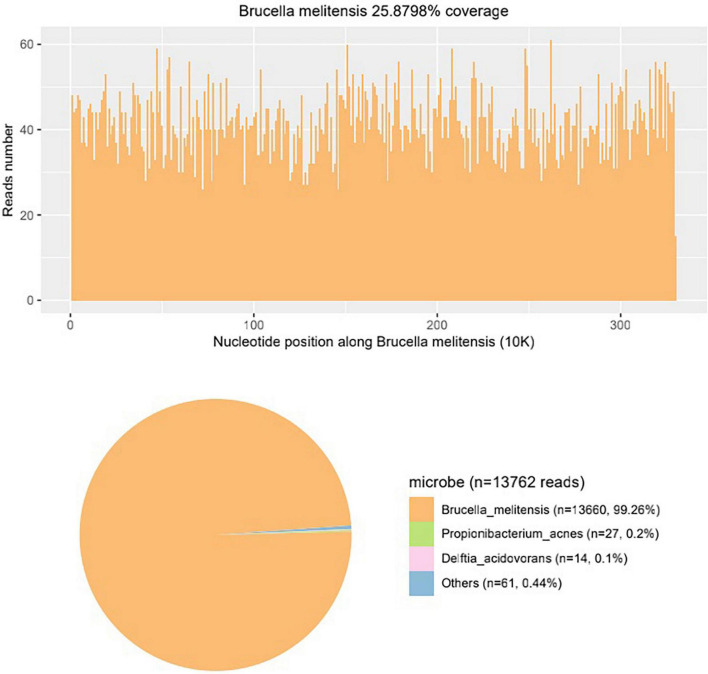
The mNGS result of the aqueous humor shows *Brucella melitensis* nucleotide reads account for the vast majority, identifying that the pathogen of the patient is *Brucella melitensis*.

Systemic steroids were suspended after admitting a series of examinations performed to identify the pathogen. General checkups were conducted: brain MRI, abdominal ultrasound, erythrocyte sedimentation rate (ESR), rheumatoid factor, antinuclear antibody spectrum. Tuberculosis bacilli, HIV, syphilis, and hepatitis pathogen detection. Meanwhile, a B-ultrasound was performed for the right eye and so was an anterior chamber punctured to extract aqueous humor for routine pathogen detection. The blood test showed elevated ESR and C-reactive protein, and the eosinophils decreased. The brain MRI showed multiple cerebral infractions, white matter demyelination changes, and brain atrophy. The abdominal ultrasound showed dense light spots in the liver and an enlarged spleen. The ocular B-scan ultrasonography showed vitreous opacity, vitreous membranes, and retinal detachment in the right eye. Meanwhile, routine pathogenic detection did not show a positive result. As there was a high degree of suspicion of infectious endophthalmitis, the aqueous humor was sent to Beijing Glantmed Medical diagnostics Lab for mNGS and cytokine detection. *Brucella melitensis* was identified by mNGS (Figure 3), and the result of cytokine detection showed a severe inflammatory reaction in the right eye. The blood titer of Brucella standard tube agglutination was 1:25, which was not too high.

From the results, a history of close contact with animals was suspected. The patient had a history of frequent exposure through raising goats, though denying consuming unpasteurized milk products or uncooked lamb. It is noticeable that the patient developed knee and waist pain 1 month after the vision loss in the right eye, and such pains passed spontaneously without treatments. Besides, 1 week before the metagenomic test results were released, her husband visited the regional hospital due to the symptoms of hip joint pain, high fever, and splenomegaly. Soon after, her husband went to the lazaretto and was diagnosed with brucellosis. Combined with the mNGS result and medical history, the patient could be diagnosed with infective endophthalmitis in the right eye, and the pathogen was *Brucella melitensis*.

**FIGURE 4:**
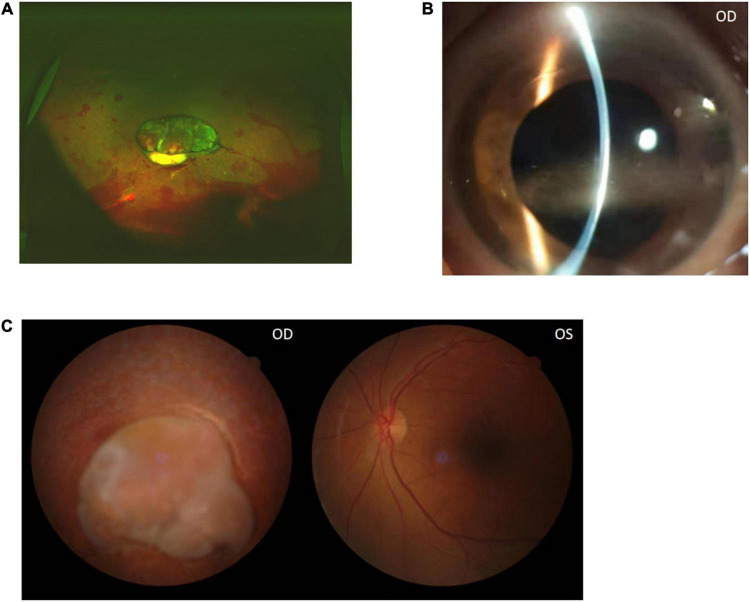
Color funds photograph after the right eye PPV surgery shows the retina contraction **(A)**. The anterior segment photograph shows cornea band-shaped degeneration 3 months later **(B)**. Color funds photograph shows there are no significant changes of the ocular fundus after 3 months **(C)**.

At the same time as the diagnosis, the patient took an oral doxycycline 10mg Bid and rifampicin 900 mg once a day, Atropine sulfate eye Gel twice a day, and Tobradex eyedrops four times a day. Due to the serious long-lasting conditions, and also for better clarification of the posterior segment, the patient underwent a lensectomy and pars plana vitrectomy (PPV) with an endolaser and silicone oil placement after 6 days of oral medication. An anterior membrane of the ciliary body in 360-degrees and extensive retinal detachment along with stiffness of the retina were noted during the surgery. The vitreous proliferating membrane adhered to the retina tightly, and these were hard to separate. Besides the extraction of the lens combined with the vitrectomy, the patient underwent 360-degree retinotomy, endolaser treatment, and silicone oil placement. While the infection of the right eye was under control, the patient was discharged from the hospital and treated with continued oral medicine (doxycycline 10 mg BID and rifampicin 900 mg a day) for 3 months (Figure 4).

The visual acuity was light perception OD and 20/30 OS at the end of oral medicine treatment, and the titer of brucella standard tube agglutination was negative. The ophthalmologic examination showed a clear cornea, quiet anterior chamber, and retina contraction. A total of 3 months after discontinuation of drugs, the cornea appeared to show band-shaped degeneration because of the effect of aqueous humor silicone oil, and there were no significant changes of the ocular fundus (Figure 4).

## Discussion

Brucellosis is a systemic disease with a wide range of clinical manifestations, and it can be diagnosed by clinical criteria and serological or culture tests. When symptoms present in organs such as the eyes (in ocular brucellosis), the condition can be misdiagnosed and patients can even remain undiagnosed (11). The first case of ocular brucellosis in human bodies was reported by Lamaire in 1924 (12). The publications that discuss the ocular involvement of brucellosis are mostly case reports from endemic regions. Uveitis (anterior, middle, and posterior) is the most common ocular manifestation, and it is usually chronic (12). Uveitis caused by brucellosis can be manifested as granulomatous or non-granulomatous, which can affect a single eye and both eyes (13). The initial corticosteroid treatment can ease the symptoms, though relapses do happen. Choroiditis can also be induced by brucella, which is usually manifested as multifocal lesions or nodular or geographic changes (14). In addition, optic neuropathy has also been found in some brucella patients, which can be manifested as optic nipple hyperemia, retrobulbar optic neuritis, papilledema, and so on (15). Other manifestations of ocular brucellosis include endophthalmitis, lacrimal gland inflammation, episcleritis, keratitis, conjunctivitis, intracranial nerve palsy, and so on (16, 17). Interestingly, in most previous reports, the manifestations of ocular brucella usually appear during the chronic phase of systemic infection (12, 14, 18). There are few cases found in the initial phases.

In this case, the symptoms of ocular panuveitis are manifested at the early stage of the patient’s disease course, and they cause serious complications such as retinal detachment and cataracts in the later stage. Currently, conventional culture methods and molecular detecting technology are popularly applied to the detection of microorganisms in endophthalmitis cases (19, 20). Nevertheless, conventional culture methods are usually ineffective, especially for slow-growing and uncommon microorganisms like brucella (21). In non-epidemic areas, it could take much time to culture and identify such microorganisms. Consequently, patients could be diagnosed late or even incorrectly. For molecular detecting technology like qPCR technology and gene chips, a limited number of pathogens can be detected merely because specific primer sets are necessary (22, 23). Compared to the methods above, mNGS is superior because it is an unbiased high-throughput sequencing approach that can theoretically detect all pathogens in a clinical sample in a short space of time (10, 24). Furthermore, mNGS can provide antibiotic resistance information by comparing genes in the organisms with those in an antibiotic resistance database (9, 24, 25). In clinic, a small amount of extracted aqueous humor from puncture under slit lamp is sufficient for mNGS, which is a low-risk and convenient method compared to extracting the vitreous humor (26). Hence, mNGS is a promising diagnostic tool for patients with difficult-to-diagnose endophthalmitis, and it provides information on antibiotic resistance and visual prognosis (24, 27).

Brucella, as an intracellular gram-negative coccobacilli, usually resides in phagocytes. Therefore, drug treatments should be implemented to avoid relapses. The current recommended dosage regimen of brucellosis involves two or more antibiotics, including doxycycline, rifampin, streptomycin, gentamicin, or trimethoprimsulfamethoxazole (28). In this case, the patient received a combination of doxycycline and rifampin, lasting for 3 months. Regrettably, the patient did not receive effective antibiotic therapy because of the negative culture result. In addition to this, the early application of corticosteroids covered up the ocular condition. Therefore, although the patient was eventually diagnosed with Brucella infection, her full vision was not preserved.

In conclusion, brucella infection of the eye as the primary manifestation is rare. Moreover, this case was diagnosed by mNGS of the aqueous humor. Both systemic and topical corticosteroids should be used cautiously when intraocular infections cannot be ruled out. Besides, while brucella has a low incidence in non-epidemic areas, it should still be considered as a probable pathogen in endophthalmitis. Most of all, the advancement of the application of mNGS in difficult-to-diagnose ocular infections can achieve early diagnosis and timely treatment to obtain a better vision outcome.

## Data Availability Statement

The original contributions presented in the study are included in the article/supplementary materials, further inquiries can be directed to the corresponding author/s.

## Ethics Statement

This study was approved by the review board of Xuzhou First People’s Hospital.

## Author Contributions

HX and LZ drafted the manuscript and collected patient information. BX edited the photographs. HL and SL critically revised the manuscript for intellectual content and supervised the project. All authors read and approved the final manuscript.

## Conflict of Interest

The authors declare that the research was conducted in the absence of any commercial or financial relationships that could be construed as a potential conflict of interest.

## Publisher’s Note

All claims expressed in this article are solely those of the authors and do not necessarily represent those of their affiliated organizations, or those of the publisher, the editors and the reviewers. Any product that may be evaluated in this article, or claim that may be made by its manufacturer, is not guaranteed or endorsed by the publisher.
